# Discovery of a new species of the genus *Stygepactophanes* from a groundwater-fed spring in southern France (Crustacea, Copepoda, Harpacticoida, Canthocamptidae)

**DOI:** 10.3897/zookeys.812.29764

**Published:** 2019-01-03

**Authors:** Diana M.P. Galassi, Frank Fiers, Marie-Josè ole-Olivier, Barbara Fiasca

**Affiliations:** 1 University of L’Aquila, Department of Life, Health and Environmental Sciences, Via Vetoio, Coppito, 67100 L’Aquila, Italy University of L’Aquila L’Aquila Italy; 2 Royal Belgian Institute of Natural Sciences, Vautierstraat 29, B-1000 Brussels, Belgium Royal Belgian Institute of Natural Sciences Brussels Belgium; 3 Université de Lyon, CNRS, UMR 5023 – LEHNA, Laboratoire d’Ecologie des Hydrosystèmes Naturels et Anthropisés, 6 rue Raphael Dubois, 69622 Villeurbanne, France Université de Lyon Lyon France

**Keywords:** Groundwater, stygobite, systematics, taxonomy, Var catchment

## Abstract

A new species of the genus *Stygepactophanes* Moeschler & Rouch, 1984 (Copepoda, Harpacticoida, Canthocamptidae) is established to accommodate a small canthocamptid population collected from a spring system in the “Parc du Mercantour”, Var catchment, southern France. The population analysed in the present study is defined by a set of morphological characters of the female, namely a very large maxilliped, a rudimentary mandibular palp, P1 with 3-segmented exopod and 2-segmented endopod, a falcate terminal claw of the P1 endopod, dorsal seta of caudal rami inserted on the inner margin, and anal operculum not overreaching the insertion of the caudal rami, thus supporting its assignment into the genus *Stygepactophanes*. The new species *Stygepactophanesoccitanus* shows marked differences with the nominotypical species of the genus that was originally described by monotypy with the species *Stygepactophanesjurassicus* Moeschler & Rouch, 1984. The main diagnostic traits of *S.jurassicus* are the absence of the P5 and a falcate outer terminal claw of P1 endopod. *Stygepactophanesjurassicus* also shows a reduced armature of the antennal exopod, bearing one seta, 1-segmented P2–P4 endopods, a reduced armature of P2–P4 exopodal segments 3 (3,4,4 armature elements, respectively), P6 bearing only one long seta, a rounded short and smooth anal operculum. Conversely the female of *S.occitanus* Galassi & Fiers, **sp. n.** has a well-developed P5, with rudimentary intercoxal sclerite, together with a falcate outer terminal claw of P1 endopod, antennal exopod bearing two elements, P4 endopod 1-segmented versus 2-segmented in P2–P3, P2–P4 exopodal segment 3 with five armature elements, P6 with three setae of different lengths, rounded anal operculum, bearing 3–4 strong spinules.

According to our present knowledge, *S.occitanus* Galassi & Fiers, **sp. n.** is assigned to the genus *Stygepactophanes* as the most conservative solution, waiting for the male to be discovered. The genus *Stygepactophanes* represents a distinct lineage within the harpacticoid family Canthocamptidae that colonised southern European groundwater, the genus being known only from the saturated karst in Switzerland and a fissured saturated aquifer in southern France. Both species of the genus are stygobites and narrow endemics, the nominotypical species being known from the type locality Source de la Doux in Délemont (Switzerland), and *S.occitanus* Galassi & Fiers, **sp. n.** described herein from a spring system of the Var catchment (France).

## Introduction

The “Parc du Mercantour” in southern France and the “Parco Naturale Alpi Marittime” in north-western Italy have promoted the development of an inventory of biological resources, including poorly known species belonging to different domains (ATBI program: All Taxa Biodiversity Inventory) and coming from different ecosystems, with special attention on the groundwater habitats. The project was also supported by the “European Distributed Institute of Taxonomy” (EDIT) (Dole-Olivier et al. 2015). The Mercantour massif has long been recognized as a European hotspot of biodiversity for both fauna and flora (Ozenda and Borel 2006, Giudicelli and Derrien 2009, Deharveng et al. 2015, Villemant et al. 2015). Its uniqueness is related to its location in Europe, where three (Alpine, Mediterranean and Continental) out of nine biogeographical regions coexist (European Environmental Agency 2018). This area is of great biogeographical interest also because of its role as a refugia during the Last Glacial Maximum, hosting a high number of narrow endemics (Biancheri and Claudin 2002, Dole-Olivier et al. 2015).

The Mercantour National Park (1465 km^2^) is situated at the south-western end of the Alpine arc. The landscape is highly diversified and defined by a complex geology (Comité de Bassin 1995a, b, c). Three major geological units are present: a central crystalline massif (granite, gneiss), external and intensively folded sedimentary formations of Secondary and Tertiary ages, and intra-Alpine thrust sheets coming from Italy and covering the subalpine zone. Groundwater is mainly represented by aquifers in fissured consolidated rocks (Comité de Bassin 1995a, b, c; Cornu et al. 2013).

In two spring mouths belonging to the same spring system out of the 27 sampled in the studied area, a small population of an unknown canthocamptid harpacticoid was discovered. The new species shows morphological affinities with *Stygepactophanesjurassicus* Moeschler & Rouch, 1984, the only species at present known for the genus. Detailed morphological analyses, and a direct comparison with the type-material of *S.jurassicus*, the type species of the genus, supported the establishment of the second species of the genus, *S.occitanus* sp. n.

## Materials and methods

Sampling was carried out according to the PASCALIS protocol, which was designed to assess groundwater biodiversity at regional scale (Malard et al. 2002). A stratified random sampling was adopted in spring-summer 2009 and in summer 2010 for the Var catchment, where 6 sampling sites were selected. Springs were sampled by using three techniques in order to maximize the sampling effort. A drift net was used to collect organisms flushed out from the aquifer by drift (Rouch et al. 1968), a Surber sample was taken to collect organisms at the surface of spring sediments and in the aquatic vegetation (Surber 1936), and a Bou-Rouch pump (Bou and Rouch 1967) was used to collect organisms at depth from the interstices of spring sediments (when present). The drift net (150 μm mesh size) was positioned at the spring outlet for eight to twelve hours (Rouch 1980). Once animals in the drift had been collected, the Surber sample was taken by moving cobbles upstream of the Surber net (150 μm mesh size) in order to dislodge animals at the surface of the spring bed sediments. Finally, sampling at depth into the spring bed sediments was carried out whenever the sediment thickness was > 30 cm. A mobile pipe was inserted into the spring bed sediments (maximum depth 50 cm below the bed surface) and 5–10 L of interstitial water and fine particles were extracted with a Bou-Rouch pump. Whatever the sampling method used, samples were elutriated, filtered through a 200-μm mesh net in the field and immediately fixed with 95% alcohol.

Only two spring mouths belonging to the Var catchment, in the Entraunes municipality, France (sites 30 and 31 in Dole-Olivier et al. (2015): Table 1, page 532), revealed the presence of two adult females of the new species collected in the drift, and three copepodids with the Surber sampling technique at the spring mouths.

**Table 1.** Female armature of P1–P4 of *Stygepactophanesoccitanus* sp. n. (female only).

	basis outer element	basis inner element	exopod	endopod
P1	+	+	I.0-I.1-II.2.0	0.0-I.1.0
P2	+	–	I.0-I.1-II.2.1	0.0-I.2.0
P3	+	–	I.0-I.1-II.2.1	0.0-I.2.0
P4	+	–	I.0-I.1-II.2.1	0.II.0

Observations and drawings were made with a phase contrast Leitz Diaplan light microscope SFZ28, equipped with a drawing tube (standard magnification 1.25×, terminal lens 18×). Morphological details were also analysed with the aid of a Leica DM 2500 interferential microscope.

The type material of *Stygepactopanesjurassicus* was also analysed. The specimens were mounted in glycerine with modelling clay dots under the cover glass; the slides were re-sealed with polyurethane varnish in the course of the present study.

Abbreviations used: Aesth: aesthetasc; P1–P6: legs 1 to 6. Armature presentation in Tables 1, 2: Roman numerals referring to spines, Arabic numerals to setae; armament position indicated as x.x.x referring to outer.distal.inner elements.

## Taxonomy

### Order Harpacticoida Sars, 1903

#### Family Canthocamptidae Brady, 1880

##### Genus *Stygepactophanes* Moeschler & Rouch, 1984

###### Type species *Stygepactophanesjurassicus* Moeschler & Rouch, 1984

####### Other species *Stygepactophanesoccitanus* Galassi & Fiers, sp. n.

######## Stygepactophanes occitanus

Taxon classificationAnimaliaHarpacticoidaCanthocamptidae

Galassi & Fiers sp. n.

http://zoobank.org/DA7D1261-22C6-4A77-B3CB-2063518C43DF

**Figure 1.**
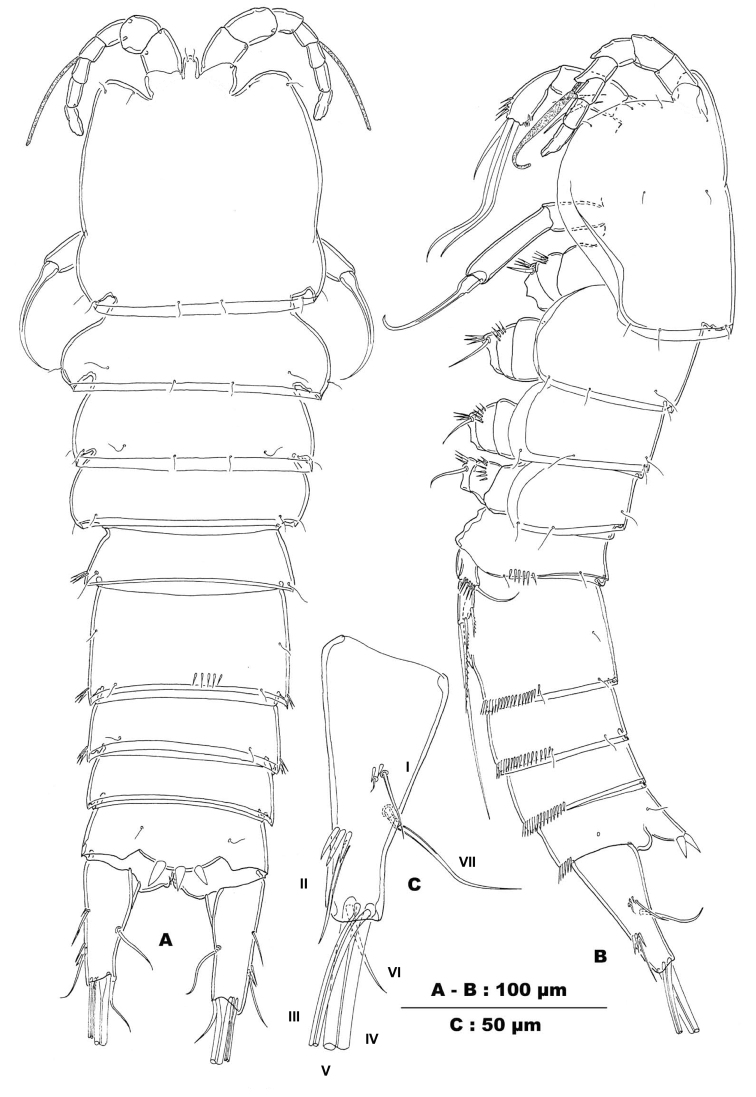
*Stygepactophanesoccitanus* sp. n. (female paratype) **A** Habitus, dorsal view **B** Habitus, lateral view **C** Left caudal ramus, outer lateral view, enlarged.

**Figure 2.**
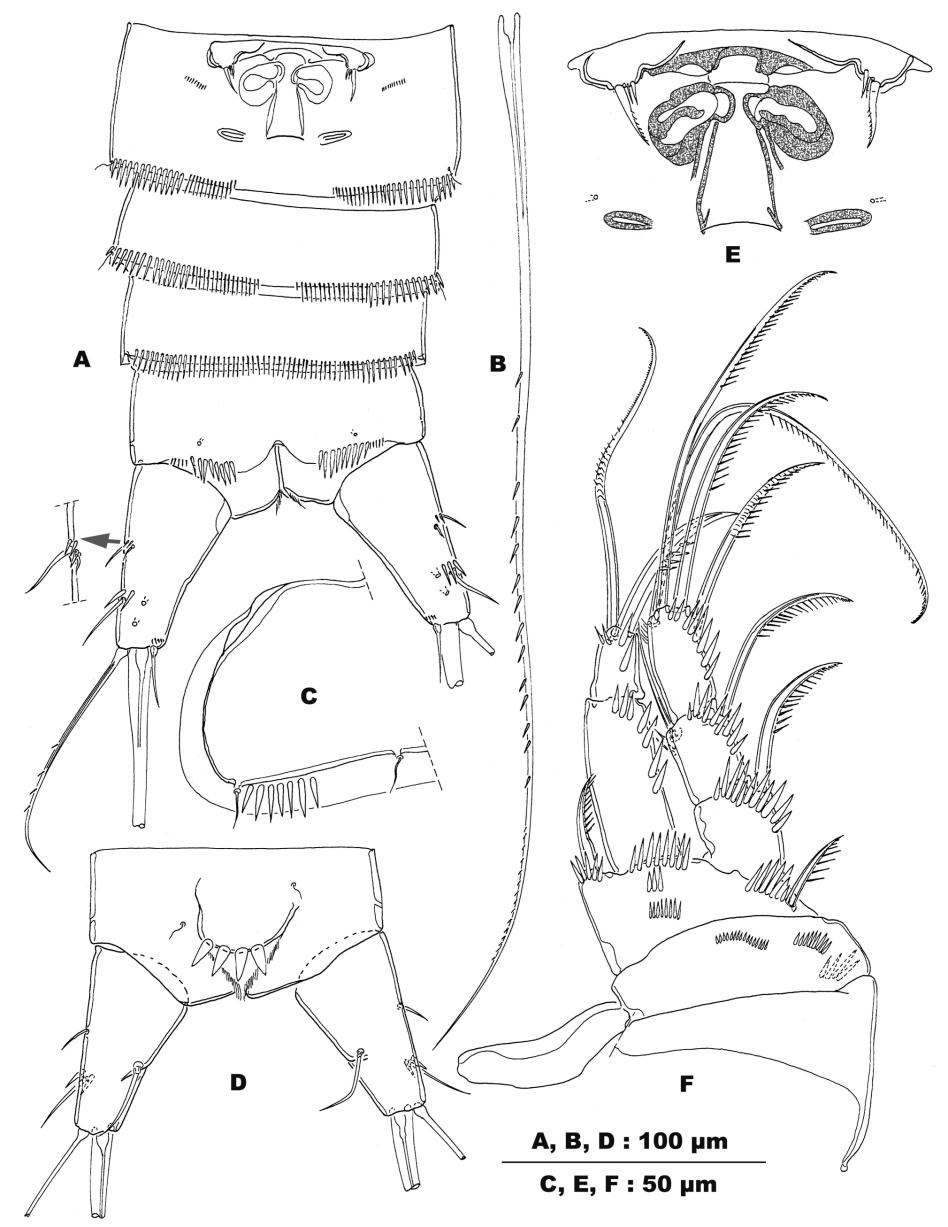
*Stygepactophanesoccitanus* sp. n. (female paratype) **A** Urosome, ventral view (arrow indicates anterolateral setae, enlarged) **B** Inner terminal seta (V) of caudal rami **C** Posteroventral edge of P4-bearing somite **D** Anal somite and caudal rami, dorsal view **E** P6 and genital complex, enlarged **F** P1, frontal.

**Figure 3.**
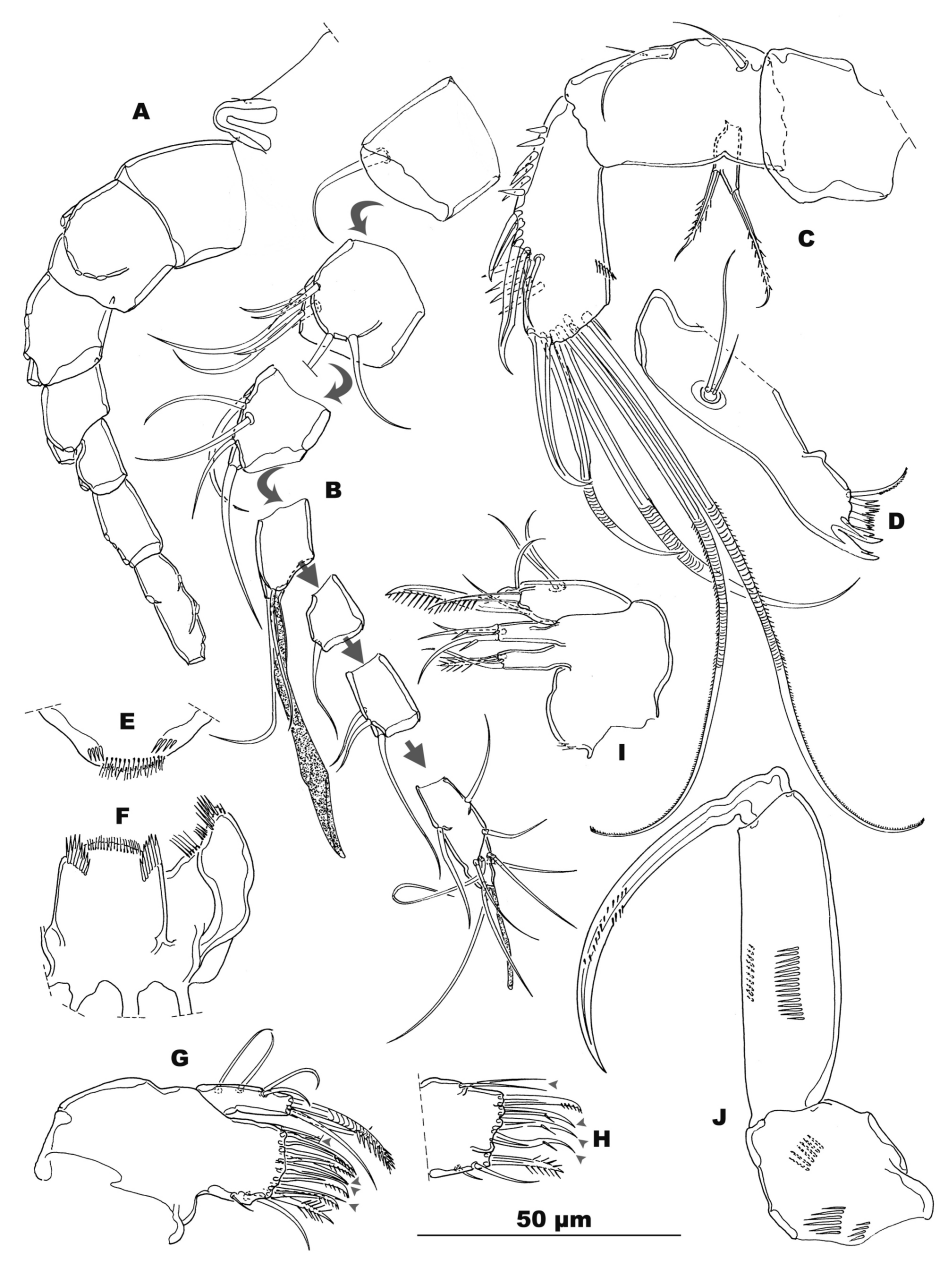
*Stygepactophanesoccitanus* sp. n. (female paratype) **A** Contour of rostrum and antennule, dorsal view **B** Antennule, exploded, armament distribution **C** Antenna **D** Mandible **E** Labium **F** Labrum **G** Maxillule, frontal view (arrows indicating elements on caudal face, see H) **H** Maxillular arthrite, caudal view (arrows indicating elements not discernable in frontal view) **I** Maxilla, frontal view **J** Maxilliped, frontal view.

**Figure 4.**
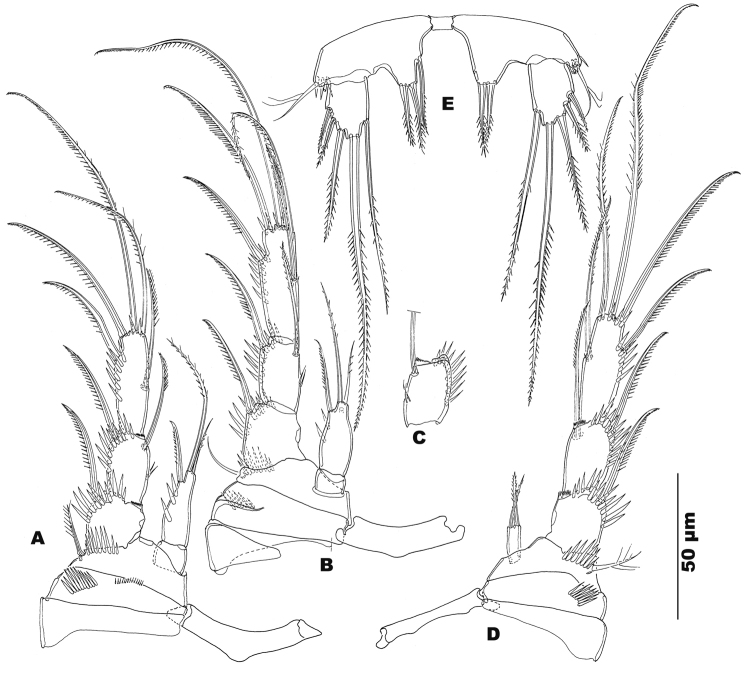
*Stygepactophanesoccitanus* sp. n. (female paratype) **A** P2, frontal view **B** P3, caudal view **C** P3, exopod 2, opposite side, frontal view **D** P4, frontal view **E** P5, caudal view.

**Figure 5.**
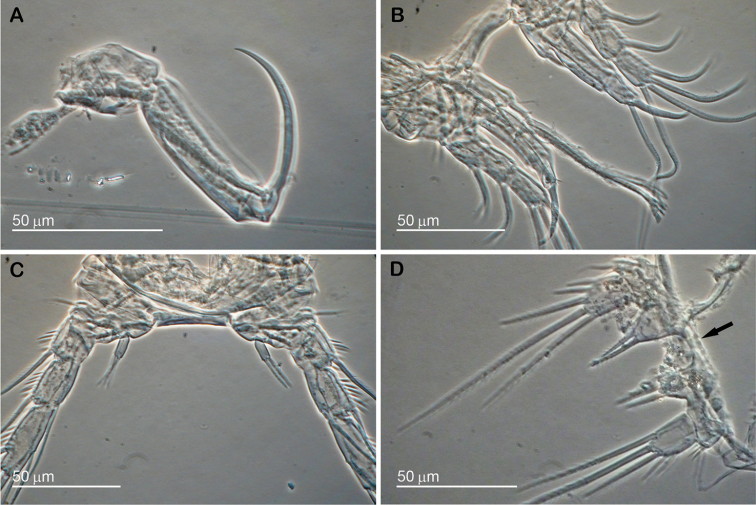
*Stygepactophanesoccitanus* sp. n. (female paratype) Optical microscopy micrographs. **A** Maxilliped **B** P1 **C** P4 **D** P5.

Figures 1 , 2 , 3 , 4 , 5

######### Material examined.

***Holotype*** here designated. Adult ♀ completely dissected and mounted in polyvinyl lactophenol on one slide, coll. M.-J. Dole-Olivier and Dominique Martin, 21 July 2009, deposited at the Muséum national d’Histoire naturelle de Paris. ***Paratypes.*** 1 ♀, same data as holotype, preserved in alcohol, coll. M.-J. Dole-Olivier and Dominique Martin, 9/08/2010. ***Additional material***: 3 ♀ copepodids collected at the Sanguinière spring system, from a spring mouth located at 2199 m above sea level.

######### Etymology.

The specific epithet refers to the region Occitania, derived from the Medieval Latin Occitania (from where the new species was collected, a region now encompassing the French administrative region Languedoc-Roussillon-Midi-Pyrénées which is located on part of the traditional Occitania and includes Roussillon).

######### Type locality.

Sanguinière spring system, Var Department, Mercantour National Park, France, Var river catchment at Entraunes municipality at 2040 m above sea level; coordinates 44.25226354N, 6.77111744E.

######### Diagnosis.

*Stygepactophanesoccitanus* Galassi and Fiers, sp. n. has a well-developed P5, with rudimentary intercoxal sclerite, together with a falcate outer terminal claw of P1 endopod, antennal exopod bearing two elements, P4 endopod 1-segmented versus 2-segmented in P2–P3, P2–P4 exopodal segment 3 with five armature elements, P6 with three setae of different lengths, rounded anal operculum, bearing 3–4 strong spinules.

######### Description of the female.

Body (Fig. 1A, B) slender and cylindrical in dorsal view, with urosome slightly narrower than prosome. Body length of holotype 425 μm, female paratype 410 μm. Podoplean flexure indistinct; prosome and urosome of same length. Integument without pits and very feeble sclerotization. Integumental windows absent; female genital and first abdominal somites completely fused forming genital double-somite; double-somite short, length/width ratio: 0.45; genital field located near anterior margin of genital somite and extended far beyond proximal half of somite. Genital complex expanded caudally to end of second third of ventral surface (Fig. 2A), with rather large reniform orifices of receptacles and long copulatory duct; the latter with wide funnel and copulatory pore; slit shaped pore present on both sides of copulatory pore.

Body ornamentation: integument of cephalothorax and urosome unornamented in the paratype, with short lateral row of spinules on left side of P4-bearing somite in the holotype (Fig. 2C); P5-bearing somite either completely smooth (holotype) or with short row of spinules on left side (paratype) (Fig. 1A, B); integument of genital double-somite either unornamented (holotype) or with short posterodorsal row of spinules (paratype; Fig. 1A); posterolateral and posteroventral margins ornamented with slender spinules, short medially (Fig. 1B), absent medioventrally (Fig. 2A); posterior margins of urosomites IV and V lateroventrally and posteroventrally with narrow spinules, interrupted mid-ventrally on urosomite IV, continuous row of spinules on urosomite V (Fig. 2A). Posterodorsal hyaline frills of body somites narrow and straight, plain. Anal somite unornamented along posterodorsal and posterolateral margins; posteroventral margin with two sets of spinules medially; outer ones minute, inner ones long and slender; anal sinus smooth, anal orifice with few slender hairs (Fig. 2A). Anal somite as long as preceding one with convex anal operculum bearing four (holotype: Fig. 2D) or three (paratype: Fig. 1A) coarse spinules along distal margin; distal margin of operculum not extending beyond anal sinus.

Caudal rami (Fig. 1A–C): conical and truncate, in both dorsal and lateral view, slightly divergent; length/width ratio: 2.75 (holotype) and 2.80 (paratype). Anterolateral accessory seta (I) minute, inserted on proximal third of caudal ramus, anterolateral seta (II) inserted on distal third of caudal ramus, accompanied by two or three long and slender spinules at insertion (Figs 1C, 2A), ca. 1.5 times longer than anterolateral accessory seta; posterolateral seta (III) slender and unornamented, outer terminal seta (IV) 1.5 times longer than ramus, sparsely serrate; inner terminal seta (V) (Fig. 2B) as long as the whole body, rather slender, sparsely serrate along outer margin (Fig. 2B); both setae IV and V without breaking plane; terminal accessory seta (VI) short, less than 1/3 length of caudal ramus; dorsal seta (VII) located at more than half of caudal ramus, near inner margin, articulating on a single basal section.

Rostrum (Figs 1A, 3A): triangular with tongue-shaped apex and apparently completely fused to cephalothorax; apex reaching just the first antennule segment; integument smooth; sensilla pair present, located subapically.

Antennule (Fig. 3A, B): rather short, backwards bent, reaching halfway along cephalothorax at most; 7-segmented, without particular integument ornamentation; armature (from proximal to distal segment): 1–8-5–2+Aesth-1–3-9+Aesth. Aesthetasc on segment IV rather wide, leaf shaped (but wrinkled in both specimens), overreaching segment VII, and fused at base with accompanying seta (acrothek); aesthetasc on segment VII slender and tongue-shaped, fused at base with terminal seta (acrothek).

Antenna (Fig. 3C): with allobasis and 1-segmented exopod; syncoxa robust, unornamented; allobasis cylindrical, 1.75 times longer than wide with two smooth setae and some slender spinules along abexopodal margin; terminal endopodal segment armed with nine elements: three lateral ones (two spines, one seta) and six distal ones (one spine, five setae); both outer distal ones fused at base; armature elements partially squamous and serrate; outer margin with two clusters of spinules; exopod located at proximal fourth of allobasis, 1-segmented and well developed, bearing one lateral and one terminal delicately serrate setae.

Mandible (Fig. 3D): rather slender, coxal gnathobase slender, without ornamentation; palp rudimentary bearing two short slender setae; cutting edge consisting of two strong bi-dentate oral and four-five multi-dentate aboral teeth.

Labrum (Fig. 3E): crescent with a subapical transverse row of spinules and an apical transverse row of setules; both edges with a short row of strong spinules.

Labium (Fig. 3F): with an oblique row of strong spinules on each side, free distal margin with setules; paragnaths armed distally with several clusters of fragile hair-like and slender spinulose elements.

Maxillule (Fig. 3G, H): well developed arthrite incorporated into praecoxa, with seven strong curved uni- or multi-serrate armature elements inserted on free distal margin, two long lateral setae and two anterior surface setae. Basis cylindrical with a total of seven elements: four naked setae on outer margin, three apical elements, one of which strong and falcate.

Maxilla (Fig. 3I): syncoxa with 2 well-developed endites that are not defined at their bases, distal endite with three setae, one serrate and two slender, bare setae; proximal endite with two setae, one bare and one serrate. Allobasis drawn out into strong unipinnate claw, rather slender, medial structure armed with widely spaced slender spinules, accompanied by one serrate and one smooth setae; endopod rudimentary, represented by two smooth setae.

Maxilliped (Figs 3J, 5A): subchelate; syncoxa short, rather quadrangular, ornamented with short rows of spinules, but lacking armature elements; basis very long (length/width ratio: 3.4) with a short row of spinules at the middle of anterior and posterior surfaces; endopod 1-segmented, extended in a long sparsely ornamented claw.

P1 (Figs 2F, 5B): prehensile; well-developed praecoxa, coxa, basis and wide intercoxal sclerite; 3-segmented exopod and 2-segmented endopod; exopod and endopod subequal in length; endopodal segment 1 quite overreaching exopodal segments 1 and 2; praecoxa and intercoxal sclerite spineless; coxa with short rows of small spinules on frontal and caudal surfaces; basis with a row of coarse spinules near articulation of each ramus, on frontal side, and near insertion of inner seta; outer seta on basis short, robust, sparsely pinnate; inner seta, bent outwards, reaching halfway along endopod 1, spiniform, serrate along outer margin only; exopod segments with coarse spinules near distal outer corner, slender spinules near insertion of inner armature elements on second and third segments; endopod with spinules along outer margin of first segment and near insertion of armature elements on second segment; armature elements of exopod serrate along outer margin only; outer terminal seta on endopodal segment 2 robust, claw-shaped, serrate midway of outer margin; inner seta geniculate, slightly serrate; armature formula as in Table 1.

P2–P4 (Figs 4A, B, D; 5C): well-developed praecoxa, coxa, intercoxal sclerite and basis; P2–P3 with 3-segmented exopods and 2-segmented endopods, P4 with 1-segmented endopod; praecoxa and intercoxal sclerite spineless, coxa and basis with short rows of spinules on anterior surface, and spineless on caudal surface; exopodal segments with coarse spinules on outer distal edge and naked inner margin except for two hair-like elements on second segment of P2 and P3; P2–P3 endopod 1 quadrate, unarmed, and spineless; P2–P3 endopodal segment 2 ca. three times longer than wide, with two or three coarse spinules along outer margin; inner margin bare; P4 endopod small, rectangular, 2.5 times longer than wide, not reaching the middle of exopodal segment 1, and unornamented; armature formula of P1–P4 exopods and endopods as in Table 1.

P5 (Figs 4E, 5D): baseoendopod and exopod not fused; baseoendopod with short inner lobe, not reaching the middle of exopod, and with short spiniform setae; left lobe with two apical and one medial setae, right lobe with two apical setae; intercoxal sclerite present, spineless; basipodal outer seta slender and short, sparsely pinnate, accompanied by cluster of spinules at insertion; exopod semi-rectangular, 1.5 times longer than wide, with five robust sparsely serrate setae: three outer, one apical, one medial; apical and medial setae the longest.

P6 (Fig. 2A, E): remnants fused, symmetrical, bearing three small setae; medial and middle setae minute and smooth, outer seta four times longer than the inner ones, robust and serrate along medial margin; legs fused medially forming a genital operculum.

######### Male.

Unknown.

###### Genus *Stygepactophanes* Moeschler & Rouch, 1984

####### Stygepactophanes jurassicus

Taxon classificationAnimaliaHarpacticoidaCanthocamptidae

Moeschler & Rouch, 1984

Figures 6 , 7

######## Material examined.

♀ labeled as “holotype” collected from “source de la Doux à Delémont” (Jura, Switzerland), 1 ♂ from “Galerie de la captage de Champ-du-Moulin”, Gorges de l’Areuse (Neufchâtel, Switzerland) without type indication; each specimen dissected with the parts mounted in glycerine. Material deposited at the Department of Arthropodology and Entomology of the Museum of Natural History of Geneva (Switzerland). The type material consists of a slide with the dissected female holotype and a slide with a dissected male; the latter without status indication and labeled to be obtained in the “Galerie de la captage de Champ-du-Moulin”. The mounts are of poor quality and many appendages appear to be absent or lost. The other specimens mentioned by Moeschler and Rouch (1984)(i.e., 2 ♂ – including 1 ♂ paratype and 1 copepodid) are missing. They seem absent in the Rouch collection and hosted at the Muséum national d’Histoire Naturelle de Paris and are certainly not present in the Genève Museum (F Fiers, pers. obs.). Fortunately, the original description by Moeschler and Rouch (1984) is detailed. The present contribution is a slight emendation of the original description, focussing on the finer morphological details, and aimed at analysing the status of the male specimen kept in Genève.

######## Supplementary description.

**Figure 6.**
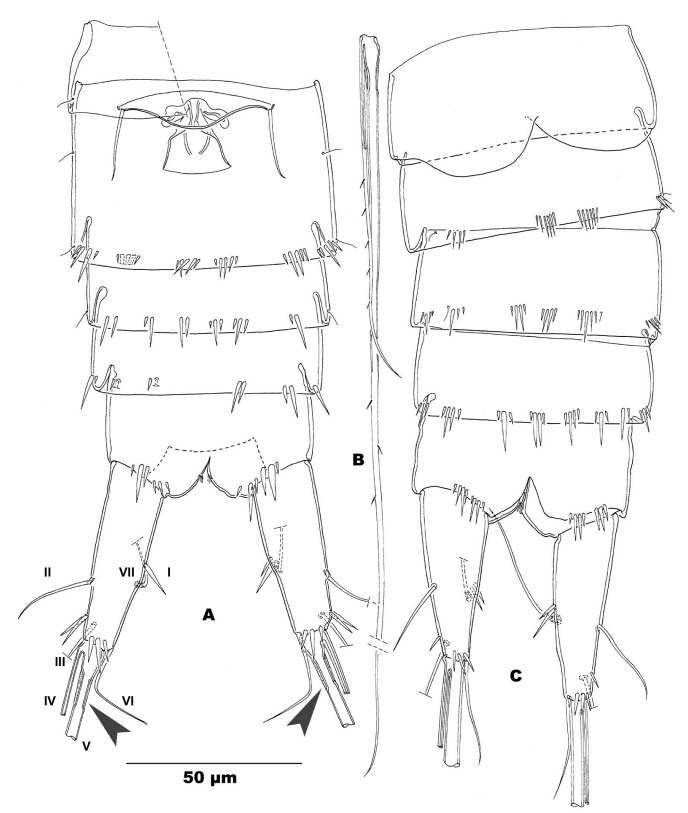
*Stygepactophanesjurassicus* Moeschler & Rouch, 1984. **A** Female urosome, ventral view (P5-bearing somite with P5 absent, left side, right side broken) **B** Female inner terminal seta (V), ventral view **C** Male urosome, ventral (**A, B** female holotype **C** male paratype).

**Female**. Urosome (Fig. 6A) without P5, urosomite I unornamented; genital double-somite short, length/width ratio: 0.73, with small receptacle orifices and wide, bell-shaped copulatory funnel and wide copulatory pore. Posterodorsal and posterolateral margins smooth; posteroventral margin with six sets of spinules of different lengths; hyaline frill absent; urosomites IV and V ornamented with six groups of spinules on posteroventral margins. Anal somite as long as preceding one, with smooth free margin of anal operculum; anal sinus not covered by operculum, smooth except for few hairs along anal orifice. Posterodorsal and posterolateral margins smooth, posteroventral margin with spinules, either long or short.

Caudal rami (Fig. 6A, C): cylindrical, only slightly enlarged at proximal part and truncate at distal part, anterolateral accessory seta (I) absent; anterolateral seta (II) inserted on distal third of caudal ramus, with some minute spinules at insertion; posterolateral seta (III) broken, accompanied by two long spinules at insertion; outer and inner terminal setae (IV–V) fused at base, both sparsely serrate and without breaking planes (Fig. 6A, B); basal part of inner terminal seta slightly inflated with narrow hyaline outer and inner membranes (outer membrane arrowed in Fig. 6A); terminal accessory seta (VI) ca. as long as half caudal rami; posteroventral margins of caudal rami with three long spinules; dorsal seta (VII) inserted at second third of caudal rami, near inner margin, articulated on basal part, and accompanied by one or two long spinules at insertion.

Antenna: with short coxa, half as long as wide, unornamented; spinules on abexopodal margin long, reaching distal fourth of allobasis; exopod with one seta, sparsely serrate along one side; endopod with distal margin bearing four elements (one spine and three setae).

**Figure 7.**
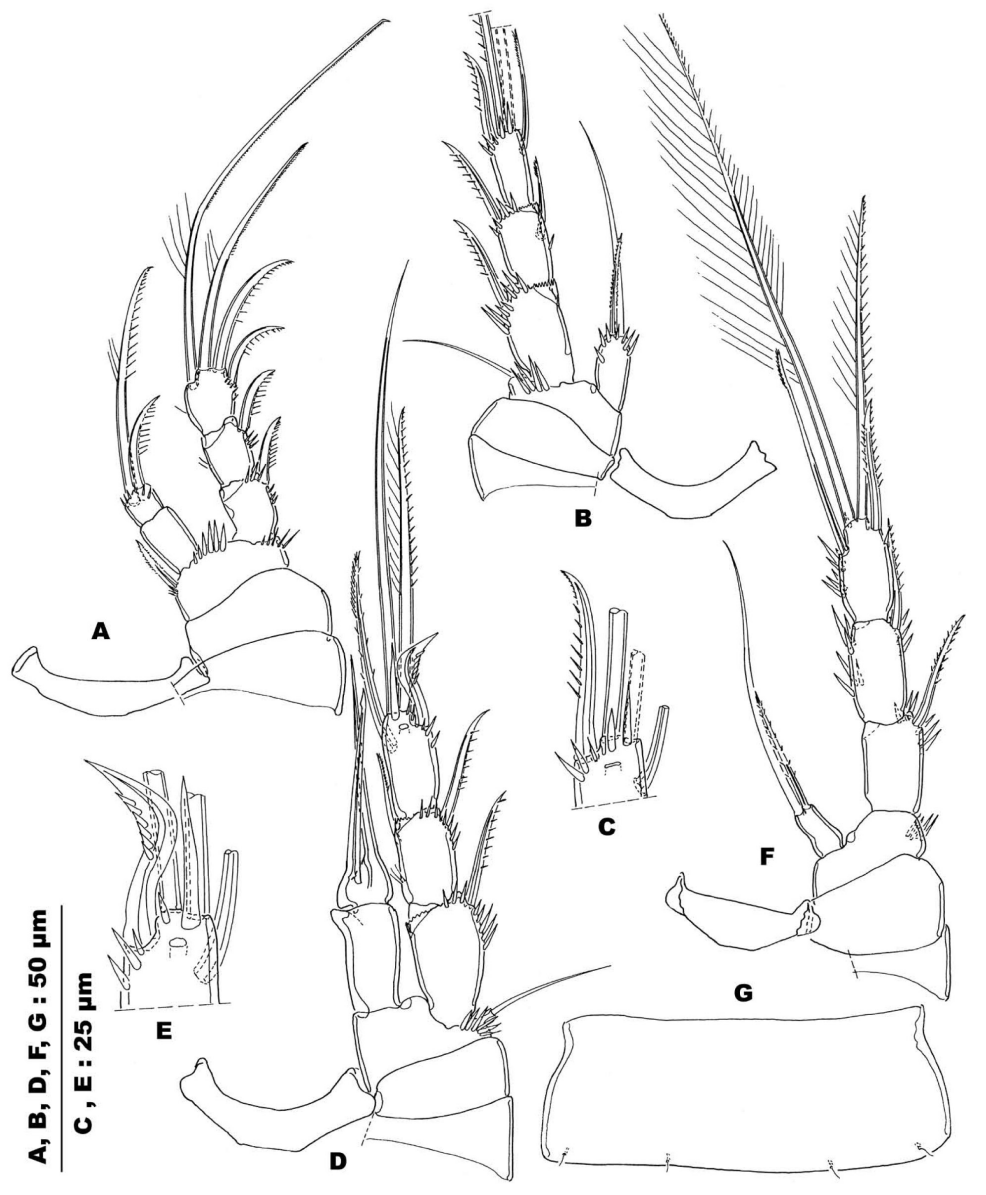
*Stygepactophanesjurassicus* Moeschler & Rouch, 1984. **A** P1, frontal **B** P3, frontal **C** P3, distal end of exopodal segment 3, enlarged **D** P3, frontal **E** P3, distal end of exopodal segment 3, enlarged **F** P4, caudal view **G** P5-bearing somite with P5 absent, ventral view (**B, C** female holotype **A, D–G**: male paratype).

P1–P4 armature as in Table 2. P3 (Fig. 7B, C): praecoxa, coxa and intercoxal sclerite well developed, unarmed and unornamented; basis with outer seta and cluster of spinules near articulation with exopod; outer elements of exopod pectinate; frontal surface of exopodal segment 3 with large subapical cuticular pore (Fig. 7C); outer element on endopod spiniform, inner one setiform.

**Table 2.** Female and male armature of P1–P4 of *Stygepactophanesjurassicus* Moeschler & Rouch, 1984 (* possible presence of two outer spines on the second segment, but likely attributable to an anomaly). Armature of female P1, P2, and P4, and male P2 taken from Moeschler and Rouch (1984).

	basis outer element	basis inner element	exopod	endopod
P1 female	–	1	I.0-I.0-II.1.1 or I.0-I.0-II.1.0	0.0-I.1.0 or 0.0-I.0.0
P1 male	–	1	I.0-I.0*-II.1.1 or I.0-I.0*-II.1.0	0.0-I.1.0
P2 female and male	–	–	I.0-I.1-I.2.0	I.1.0
P3 female	+	–	I.0-I.1-I.2.1	I.1.0
P3 male	+	–	I.0-I.1-I.2.1	0.0-modified
P4 female and male	–	–	I.0-I.0-I.2.1	I.1.0

P5 absent.

P6 (Fig. 6A): reduced, represented by a single (smooth?) long seta, and confluent midventrally forming a caudally expanded convex plate covering anterior part of genital field.

**Male.** Urosome (Fig. 6C): urosomite I without P5, unornamented (Fig. 7G); urosomites II–V ornamented (urosomites III–V as in female, and urosomite II ornamented as urosomites III–V); outer terminal and inner terminal setae (IV–V) of caudal rami fused at base; seta V not inflated and lacking hyaline membranes; dorsal seta (VII) inserted near or on inner margin of caudal rami.

P1 (Fig. 7A): praecoxa, coxa and intercoxal sclerite unarmed and unornamented; intercoxal sclerite narrow and wide; medial margin of exopodal segments 2 and 3 with sparse hairy ornament; outer margins of exopodal segments with few spinules; armature elements of inner margin and inner distal margin delicately serrate outwardly, plumose midway inwardly; endopodal segment 1 without spinule ornament, endopodal segment 2 with spinules along distal margin; outer terminal element on endopodal segment 2 claw-shaped (falcate), serrate along outer margin; inner element robust with spinular appearance, outwardly serrate, plumose midway inwardly, and at least twice as long as outer element; left and right legs identical.

P3 (Fig. 7D, E) with well-developed praecoxa, coxa and intercoxal sclerite, all unarmed and unornamented; basis as in female; medial armature element on exopodal segment 3, segment more robust than in female; outer spine on exopodal segment 3 robust, claw-shaped and strongly serrate in middle of outer margin; apical margin with long median spinule and wide subapical pore on frontal surface (Fig. 7E); endopod 2-segmented; proximal segment twice as long as wide, with medial distal corner forming a truncate expansion; distal segment globular and extended into two equally long sharp apophyses overreaching exopod.

P4 (Fig. 7F) with well-developed praecoxa and coxa; intercoxal sclerite unarmed and unornamented; basis without outer seta and with short row of spinules near outer margin; medial element on exopodal segment 3 with distal inner margin pectinate; endopod 1-segmented, twice as long as wide, with outer terminal element spiniform and inner one setiform.

P5 absent.

P6 (Fig. 6C) represented as a caudally symmetrical bilobate plate, without setae and completely smooth.

Moeschler and Rouch (1984) reported the aberrant nature of the exopodal armature of P1 in the male specimen collected at the “Captage de Champ-du-Moulin”. They provided an illustration (Moeschler and Rouch (1984): fig. 7b, page 968) of a leg with two spines on exopodal segment 2, and only three armature elements on its terminal segment. The opposite leg was mentioned as being armed in the same way as described for the female holotype with one outer spine on exopodal segment 2 and four elements on the terminal segment.

Re-examination of the slide kept at Genève labeled: “Galerie de la Captage de Champ-du-Moulin, Gorges de l’Areuse (NE); 17.11.1981” revealed, however, that both legs are identical, and resemble the female P1 as illustrated in Moeschler and Rouch (1984): fig. 5a, page 965. This observation confirms that the male paratype deposited at Genève must have been mislabeled during processing of the slides.

###### Key to the species of the genus *Stygepactophanes* (based on females only)

**Table:** 

1	P1 endopodal segment 1 ca. 2 times longer than endopodal segment 2, slightly overreaching exopodal segment 1; P2–P4 endopods 1-segmented, P5 absent, caudal rami cylindrical and long (length/width ratio: 3.3–3.5), anal operculum rounded and smooth	***Stygepactophanesjurassicus* Moeschler & Rouch, 1984**
–	P1 endopodal segment 1 ca. 3 times longer than endopodal segment 2, quite overreaching exopodal segment 2; P2–P3 endopods 2-segmented, P4 endopod 1-segmented, P5 well developed, with rudimentary intercoxal sclerite, caudal rami subconical and long (length/width ratio: 2.54), anal operculum with strong spinules	***Stygepactophanesoccitanus* sp. n.**

## Remarks

*Stygepactophanesoccitanus* sp. n. does not fit the diagnosis of any defined genus in the keys available to date (Lang 1948, Borutzky 1952, Dussart and Defaye 1995, 2001, Boxshall and Halsey 2004) and led us to the genus *Epactophanes* Mrázek, 1893 using the identification keys of Wells (2007). In more detail, *S.occitanus* sp. n. could be placed among the genera unified in Borutzky’s (1952) subfamily Epactophaninae Borutzky, 1952 currently including *Epactophanes* and *Epactophanoides* Borutzky, 1966 (and eventually *Ceuthonectes* Chappuis, 1924, see Dussart and Defaye 2001, and below).

There are several indications that *S.occitanus* sp. n. is an obligate groundwater species (as well as *S.jurassicus*). The fine integument almost completely devoid of ornamentation, the body transparency and the absence of eye pigmentation, the large and wide antennule main aesthetasc and the reduced appendages are relevant stygomorphic traits. Moreover, the presence of the new species in the outflow of two spring mouths fed by the same aquifer, and its low abundance (no other specimens were found in additional samplings (M-J Dole-Olivier and D Martin, pers. comm.) are supplementary arguments to support this contention.

Obligate groundwater canthocamptids such as members of *Stygepactophanes*, *Lessinocamptus* Stoch, 1997, *Spelaeocamptus* Chappuis, 1933, and *Paramorariopsis* Brancelj, 1991, among others, are known to have a limited distribution, and in most cases are narrow endemics (Galassi 1997, Fiers and Moldovan 2008, Galassi et al. 2009, Brancelj 2009, 2011, Di Lorenzo et al. 2018). Their affinities with the epigean members of the family often remain obscure. Apparently they represent local derived strays displaying adaptations, mostly in terms of reduction and/or characters’ losses. These reductions and/or characters’ losses are frequently found in obligate groundwater species of other harpacticoid families, such as Ectinosomatidae, Ameiridae, Rotundiclipeidae, and Leptopontiidae and even in some stygobiotic cyclopoid genera (Galassi et al. 1999a, b, Galassi and De Laurentiis 2004a, b).

As far as the stygobiotic Canthocamptidae are concerned, although their roots have different origins in the evolutionary history of the family, they share remarkable similarities such as simplified body shape, delicate integument, long and widened main antennule aesthetasc, short P1 endopods with prominent falcate terminal claw (even more conspicuous in *Stygepactophanes* than in *Lessinocamptus* and *Elaphoidella* Chappuis, 1929), and reduction in mouthparts and swimming legs, likely as a result of adaptive convergence by means of heterochrony (Galassi et al. 1999a, b, Galassi et al. 2009). The very long maxilliped shared by *S.jurassicus* and *S.occitanus* sp. n. may either be considered an autapomorphy of the genus, or the result of adaptation to a similar trophic niche (adaptive trait?). Actually long maxillipeds are present also in members of other harpacticoid families (Galassi and De Laurentiis 2004b), as in the Parastenocarididae (e.g., *Simplicarislethaea* Galassi and De Laurentiis, 2004, and in *Parastenocarisandreji* Brancelj, 2000) suggesting also that this character may have appeared more than once in the evolutionary history of the Harpacticoida.

Relationships between *Stygepactophanes* and the *Epactophanes*-*Epactophanoides* lineage, as might be assumed, cannot be substantiated as they do not share the particular caudally displaced female genital complex, as found in *Stygepactophanes* and the topology of the dorsal seta on caudal rami inserted near or on the inner margin of caudal rami (considered herein an autapomorphy of the *Stygepactophanes* lineage).

*Stygepactophanesjurassicus* and *S.occitanus* sp. n. share similar habitus, long maxilliped, P1 with falcate outer apical element, P1 endopodal segment 1 from 2 to 3.5 times longer than endopodal segment 2, topology and development of the genital field in the female extending at least to the proximal half of the genital double-somite, and the inner position of the dorsal seta of the caudal rami.

Nevertheless, the females are clearly distinguishable on the basis of the following characters: antennal exopod with one seta in *S.jurassicus* versus two in *S.occitanus* sp. n.; mandibular palp 1-segmented in *S.jurassicus* but absent in *S.occitanus* sp. n., where only two remnant setae are present, 1-segmented P2–P3 endopods in *S.jurassicus* versus 2-segmented in *S.occitanus* sp. n.; P2 exopodal segment 3 with three elements in *S.jurassicus* versus five elements in *S.occitanus* sp. n., P3–P4 exopodal segment 3 with four elements in *S.jurassicus* versus five elements in *S.occitanus* sp. n.; P5 absent in *S.jurassicus* but present and well developed in *S.occitanus* sp. n.; P6 with one outer long seta in *S.jurassicus* versus three setae, the outer the longest in *S.occitanus* sp. n.; anal operculum rounded and smooth in *S.jurassicus* versus ornamented by strong spinules in *S.occitanus* sp. n. The affinities of *S.occitanus* sp. n. and *S.jurassicus* are indisputable, and the main difference relies on the primitive character states shown by *S.occitanus* sp. n.; namely the well-developed P5, the 2-segmented P2–P3 endopods, and a higher number of armature elements of the exopodal segment 3 of P2–P4.

To our present knowledge, and on the basis of the missing information about the male of *S.occitanus* sp. n., the assignment of the new species to the genus *Stygepactophanes* is the most conservative solution. Pending the discovery of the male, an emended diagnosis is provided based on females only.

## Emended diagnosis of the genus *Stygepactophanes*

Canthocamptidae. Small canthocamptid with cylindrical body without clear demarcation between prosome and urosome; integument without pits and very feeble sclerotization. Eyeless. Integumental windows absent; female genital and first abdominal somites completely fused forming a genital double-somite; genital field located near anterior margin of genital somite and developed at least as far as the middle length of the same somite. Body ornamentation: integument of cephalothorax and urosome unornamented. Posterodorsal frills of body somites narrow and straight, plain; anal somite unornamented along posterodorsal and posterolateral margins. P5-bearing somite either completely smooth or with short row of spinules; integument of genital double-somite either unornamented or with short posterodorsal row of spinules; posterolateral and posteroventral margins ornamented with slender spinules, short medially, absent medioventrally. Operculum not protruding beyond the insertion of caudal rami, rounded and smooth or bearing strong spinules. Caudal rami cylindrical or conical, elongated, bearing seven setae or missing the anterolateral accessory seta (I); dorsal seta inserted near or on inner margin of caudal ramus. Rostrum small, not defined at base; antennule 7-segmented with main aesthetasc on segment IV very large; antenna allobasis with two abexopodal setae and 1-segmented exopod bearing one or two setae; mandibular palp rudimentary, represented by a small segment bearing one short seta, or represented by two setae only; maxillule and maxilla reduced, the latter with 2 endites; maxilliped very long, clearly discernible in dorsal view with a long and thin basis and falcate endopod; P1–P4 with 3-segmented exopods; P1–P3 with 1- or 2-segmented endopods, P1 with 2-segmented endopod, falcate and bearing a serrate claw; endopodal segment 1 always shorter than endopodal segment 2; P4 with 1-segmented endopod; P5 absent or present and well developed; in the latter case, intercoxal sclerite present.

## Discussion

Moeschler and Rouch (1984) faced problems in allocating *S.jurassicus* among the canthocamptids, and resurrected Borutzky’s (1952) family subdivision in subfamilies. Fortunately, they have had the chance to collect and analyse the male. *Stygepactophanes* was assumed to be related either to the Morariinae Borutzky, 1952 or to the Epactophaninae Borutzky, 1952, with a certain bias towards the latter, an opinion that has not been challenged so far. Borutzky’s (1952) subfamilies have not elicited much support and have been generally ignored (Wells 2007), with a few exceptions (Dussart and Defaye 1995, 2001), and currently partially re-introduced by Walter and Boxshall (2018). The validity of these canthocamptid subfamilies is still debated and in need of revision.

That the subfamily Epactophaninae with the only genera *Epactophanes* Mrázek, 1893 and *Epactophanoides* Borutzky, 1966 may constitute a natural group has some ground based on the unique male P3 endopod and the morphology of the female genital complex. The proposal that *Stygepactophanes*, *Ceuthonectes* and *Ligulocamptus* Guo, 1998 (as suggested in Dussart and Defaye 1995, 2001) could be considered affiliated members is questionable. *Ceuthonectes* and *Stygepactophanes* do not display any of the diagnostic features shared by *Epactophanes* and *Epactophanoides* (i.e., the sole male P3 endopod and the female genital complex) and should reasonably be placed outside the epactophanid lineage. *Ligulocamptus* is apparently close to *Mesochra* Boeck, 1864 as proposed by Guo (1998) although this statement requires confirmation in order to establish their real affinities.

The alternative in which *Stygepactophanes* may enter the diagnosis of the Morariinae can only be partially supported. Borutzky (1952) originally included the genera *Moraria* T. and A. Scott, 1893, *Morariopsis* Borutzky, 1931 and *Ceuthonectes* in this subfamily. The group has gradually been expanded with the addition of *Pseudomoraria* Brancelj, 1994, *Paramorariopsis*, *Gulcamptus* Miura, 1969 and *Itunella* Brady, 1896 (see Dussart and Defaye 2001). Neither *Ceuthonectes* nor any of the recently added taxa are directly related to *Moraria* and *Morariopsis* as they are representatives of different lineages in the Canthocamptidae (Fiers and Jocque 2013).

However, *Stygepactophanes* displays some remarkable similarities with *Ceuthonectes*. In the latter, the sexually dimorphic P3 endopod is the main morphological trait shared with *Stygepactophanes*. Dimorphic traits of the male P2 and P4 endopods are limited (length/width of the segment, fusion of segments) and both legs have the same number of armature elements in males and females. In contrast, the male P3 endopod, 2-segmented in both sexes, is distinctly modified. The proximal segment is enlarged with one or more reinforcements of the proximal margin, the distal segment is quite short, has a globular aspect, and bears two narrow and spiked lanceolate armature elements. Moreover, the terminal segment of the male P3 possesses a long hyaline tubular expansion of the frontally located pore (originally interpreted as a spinule by Chappuis (1924)), a structure absent in the female. The dimorphic aspects of the endopods in *S.jurassicus* (the only species of the genus for which the male is known) are very similar to those of *Ceuthonectes*, but the tubular structure on the frontal surface of the terminal exopodal segment is absent. However, in *S.jurassicus* one of the spinules along the distal margin is conspicuously longer than in the female (see Fig. 7C, E). Although these terminal structures are not identical, their topology on (in *Ceuthonectes*) and near (in *Stygepactophanes*) the frontal pore supports the hypothesis that they may be homologous and likely have a similar function.

## Conclusions

*Stygepactophanesoccitanus* sp. n. is assigned to the genus *Stygepactophanes*. The new species shows several morphological characters in a primitive state, if compared to the type species of the genus *S.jurassicus*, weakening the attribution of the new species to a new genus, albeit closely related to *Stygepactophanes*. The morphological affinities of this genus to the other genera of the family Canthocamptidae have generated doubts since its original description. We have postulated that the genera *Ceuthonectes* and *Stygepactophanes* may represent a divergent lineage within the Canthocamptidae. Unfortunately, because of the complex systematics of the family still being in a state of flux, the relationships of this lineage to other members of the family remain unresolved. Presumably, *Stygepactophanes* entered the groundwater a very long time ago in the evolutionary history of the family Canthocamptidae, and has no representatives in surface waters (phylogenetic and distributional relict), as in the case of other harpacticoid genera, as well as the entire copepod order Gelyelloida.

## Supplementary Material

XML Treatment for Stygepactophanes occitanus

XML Treatment for Stygepactophanes jurassicus

## References

[B1] Biancheri J-Y Claudin J (2002) Atlas des Parcs nationaux de France, Parc national du Mercantour. In: Biancheri J-Y and Claudin J (Eds) Parc national du Mercantour.Ministère de l’Écologie et du Développement durable, MEDD, Nice, 1–80.

[B2] Borutzky EV (1931) Materialien zur Harpacticidenfauna des Baikalsees. II. Zoologischer Anzeiger 93: 263–273.

[B3] Borutzky EV (1952) Fauna USSR. Crustacea, Volume III, No. 4. Freshwater Harpacticoida. Izdaniya Akademia Nauk SSSR 50, 396 pp. [English translation by A Mercado, 1964, published by the Israel Program for Scientific Translation, Jerusalem]

[B4] Borutzky EV (1966) Copepoda peshcher Primorskogo Kraya. Copepoda of the caves of the marine territory (Primorski Krai). Zoologicheskii Zhurnal 45: 770–772. [Russian with English summary]

[B5] Bou C Rouch R (1967) Un nouveau champ de recherches sur la faune aquatique souterraine. Comptes rendus de l’Académie des Sciences de Paris 265: 369–370.

[B6] Boxshall GA Halsey SH (2004) An introduction to copepod diversity. Volume 1. The Ray Society, London 166: 1–421.

[B7] Brady GS (1880) A monograph of the free and semi-parasitic Copepoda of the British Islands. Ray Society 3: 1–83.

[B8] Brady GS (1896) On Entomostraca collected in the Solway district and the Seaton sluice, Northumberland, during the summer of 1894. Natural History Transactions of Northumberland 13: 19–33.

[B9] Brancelj A (1991) *Paramorariopsisanae* gen. n., sp. n. and the female of *Ceuthonectesrouchi* Petkovski, 1984: two interesting harpacticoids (Copepoda: Crustacea) from caves in Slovenia (NW Yugoslavia). Stygologia 6: 193–200.

[B10] Brancelj A (1994) *Pseudomorariatriglavensis* gen. n., sp. n. (Copepoda, Harpacticoida) from a high-alpine reservoir in Slovenia. Hydrobiologia 294: 89–98. 10.1007/BF00016848

[B11] Brancelj A (2000) *Parastenocarisandreji* n. sp. (Crustacea; Copepoda) – the first record of the genus in Slovenia (SE Europe). Hydrobiologia 437: 235–239. 10.1023/A:1026513917821

[B12] Brancelj A (2009) Fauna of an unsaturated karstic zone in central Slovenia: two new species of Harpacticoida (Crustacea: Copepoda), *Elaphoidellamillennii* n.sp. and *E.tarmani* n. sp., their ecology and morphological adaptations. Hydrobiologia 621: 85–104. 10.1007/s10750-008-9634-3

[B13] Brancelj A (2011) Copepoda from a deep-groundwater porous aquifer in contact with karst: description of a new species, *Paramorariopsisbrigitae* n. sp. (Copepoda, Harpacticoida). Crustaceana, Studies on Freshwater Copepoda, 85–104.

[B14] Chappuis PA (1924) Descriptions préliminaires de Copépodes nouveaux de Serbie. Buletinul Societatii de Stiinte din Cluj, România 2: 27–45.

[B15] Chappuis PA (1929) Révision du genre *Canthocamptus* Westwood (Note préliminaire). Buletinul Societatii de Stiinte din Cluj 4: 41–50.

[B16] Chappuis PA (1933) Copépodes (première série). Avec l’énumération de tous les Copépodes cavernicoles connus en 1931. Archives de Zoologie Expérimentale et Générale 76: 1–57.

[B17] Comité de Bassin RMC (1995a) Atlas du Bassin Rhône Méditerrannée Corse. 3 – Eaux souterraines. Territoire de Haute Durance. http://sierm.eaurmc.fr/sdage/documents/A16_3.pdf

[B18] Comité de Bassin RMC (1995b) Atlas du Bassin Rhône Méditerrannée Corse. 3 – Eaux souterraines. Territoire côtiers alpins Est. http://sierm.eaurmc.fr/sdage/documents/A21_3.pdf

[B19] Comité de Bassin RMC (1995c) Atlas du Bassin Rhône Méditerrannée Corse. 3 – Eaux souterraines. Territoire Verdon. http://sierm.eaurmc.fr/sdage/documents/A19_3.pdf

[B20] Cornu J-F Eme D Malard F (2013) The distribution of groundwater habitats in Europe. Hydrogeology Journal 21(5): 949–960. 10.1007/s10040-013-0984-1

[B21] Deharveng L Bedos A Daugeron C Villemant C Judson MLI (2015) Organization, usefulness and limitations of an ATBI (All Taxa Biodiversity Inventory): the inventory of terrestrial invertebrates in the Mercantour National Park. In: Daugeron C Deharveng L Isaia M Villemant C Judson M (Eds) Mercantour/Alpi Marittime All Taxa Biodiversity Inventory.Zoosystema 37(1), 9–30. 10.5252/z2015n1a1

[B22] Di Lorenzo T Cipriani D Fiasca B Rusi S Galassi DMP (2018) Groundwater drift monitoring as a tool to assess the spatial distribution of groundwater species into karst aquifers. Hydrobiologia 813: 137–156. 10.1007/s10750-018-3515-1

[B23] Dole-Olivier M-J Galassi DMP Fiers F Malard F Martin P Martin D Marmonier P (2015) Biodiversity in mountain groundwater: the Mercantour National Park (France) as a European hotspot. In: Daugeron C Deharveng L Isaia M Villemant C Judson M (Eds) Mercantour/Alpi Marittime All Taxa Biodiversity Inventory.Zoosystema 37: 529–550. 10.5252/z2015n4a1

[B24] Dussart B Defaye D (1995) Introduction to the Copepoda. Guides to the Identification of Microinvertebrates of Continental Waters of the World 7: 1–277.

[B25] Dussart B Defaye D (2001) Introduction to the Copepoda (2^nd^ edn). Guides to the identification of Microinvertebrates of continental waters of the world 16: 1–344.

[B26] European Environmental Agency (2018) European Environmental Agency. https://www.eea.europa.eu/data-and-maps/data/biogeographical-regions-europe-3 [accessed on 10/09/2018]

[B27] Fiers F Jocque M (2013) Leaf litter copepods from a cloud forest mountain top in Honduras (Copepoda: Cyclopidae, Canthocamptidae). Zootaxa 3630: 270–290. 10.11646/zootaxa.3630.2.4 26131511

[B28] Fiers F Moldovan OT (2008) Redescription of *Spelaeocamptusspelaeus* (Chappuis, 1925), a subterranean copepod endemic to the Apuseni Mountains in Romania (CopepodaHarpacticoida). Subterranean Biology 6: 51–64.

[B29] Galassi DMP (1997) Little known harpacticoid copepods from Italy, and description of *Parastenocariscrenobia* n. sp. (Copepoda, Harpacticoida). Crustaceana 70: 694–709. 10.1163/156854097X00131

[B30] Galassi DMP De Laurentiis P (2004a) Little-known cyclopoids from groundwater in Italy: re-validation of *Acanthocyclopsagamus* and redescription of *Speocyclopsitalicus* (Crustacea, Copepoda, Cyclopoida). Vie et Milieu 54: 203–222.

[B31] Galassi DMP De Laurentiis P (2004b) Towards a revision of the genus *Parastenocaris* Kessler, 1913: establishment of *Simplicaris* gen. n. from groundwaters in Central Italy and review of the *brevipes*-group (Copepoda, Harpacticoida, Parastenocarididae). Zoological Journal of the Linnean Society 140: 417–436. 10.1111/j.1096-3642.2003.00107.x

[B32] Galassi DMP De Laurentiis P Dole-Olivier M-J (1999a) *Nitocrellopsisrouchi* sp. n., a new ameirid harpacticoid from phreatic waters in France (Copepoda: Harpacticoida: Ameiridae). Hydrobiologia 412: 177–189. 10.1023/A:1003855120904

[B33] Galassi DMP Dole-Olivier M-J De Laurentiis P (1999b) Phylogeny and biogeography of the genus *Pseudectinosoma*, and description of *P.janineae* sp. n. (Crustacea: Copepoda, Ectinosomatidae). Zoologica Scripta 28: 289–303. 10.1046/j.1463-6409.1999.00018.x

[B34] Galassi DMP Huys R Reid JW (2009) Diversity, ecology and evolution of groundwater copepods. Freshwater Biology 54: 691–708. 10.1111/j.1365-2427.2009.02185.x

[B35] Giudicelli J Derrien M (2009) Les invertébrés des eaux courantes du Parc national du Mercantour: inventaire, biogéographie et écologie. I Trichoptères, Diptères Blépharicérides et Simuliides. Ephemera 10(1): 43–69.

[B36] Guo XM (1998) *Ligulocamptusloffleri* n.g., n.sp. (Copepoda: Harpacticoida) from Chengdong Lake in China. Hydrobiologia 368: 209–215. 10.1023/A:1003218720209

[B37] Lang K (1948) Monographie der Harpacticiden. Håkan Ohlsson, Lund, 2 vols, 1682 pp.

[B38] Malard F Dole-Olivier M-J Mathieu J Stoch F (2002) Sampling manual for the assessment of regional groundwater biodiversity. http://www.pascalis-project.com

[B39] Miura Y (1969) Results of the speleological survey of South Korea, 1966. XIV. Subterranean harpacticoid copepods of South Korea. Bulletin of the National Science Museum, Tokyo, Series A, Zoology 12: 241–254.

[B40] Moeschler P Rouch R (1984) Un nouveau genre de Canthocamptidae (Copepoda, Harpacticoidea) des eaux souterraines de Suisse. Revue Suisse de Zoologie 91: 959–972. 10.5962/bhl.part.81592

[B41] Mrázek A (1893) Beitrag zur Kenntis der Harpacticidenfauna des Süsswassers. Zoologische Jahrbücher (Syst.) 7: 89–130.

[B42] Ozenda PG Borel JL (2006) Végétation des Alpes sudoccidentales. Un sommet de la biodiversité. Braun-Blanquetia 41: 1–45.

[B43] Rouch R (1980) Les Harpacticides, indicateurs naturels de l’aquifère karstique. Mémoires Société géologique de France, Hors-Série 11: 109–116.

[B44] Rouch R Juberthie-Jupeau L Juberthie C (1968) Recherches sur les eaux souterraines – Essai d’étude du peuplement de la zone noyée d’un karst. Annales de Spéléologie 23: 717–733.

[B45] Sars GO (1903) CopepodaHarpacticoida. Parts I–II, Misophriidae, Longipediidae, Cerviniidae, Ectinosomidae (part). An Account of the Crustacea of Norway, with short descriptions and figures of all the species. Bergen Museum 5: 1–28.

[B46] Scott T Scott A (1893) On some new or rare Scottish Entomostraca. Annals and Magazine of Natural History ser 6, 11: 210–215. 10.1080/00222939308677499

[B47] Stoch F (1997) A new genus and two new species of Canthocamptidae (Copepoda, Harpacticoida) from caves in northern Italy. Hydrobiologia 350: 49–61. 10.1023/A:1003072813906

[B48] Surber EW (1936) Rainbow trout and bottom fauna production in one mile of stream. Transactions of the American Fisheries Society 66: 193–202. 10.1577/1548-8659(1936)66[193:RTABFP]2.0.CO;2

[B49] Villemant C Daugeron C Gargominy O Isaia M Deharveng L Judson MLI (2015) The Mercantour/Alpi Marittime All Taxa Biodiversity Inventory (ATBI): achievements and prospects. In: Daugeron C Deharveng L Isaia M Villemant C Judson M (Eds) Mercantour/Alpi Marittime All Taxa Biodiversity Inventory.Zoosystema 37(4), 667–679. 10.5252/z2015n4a10

[B50] Walter TC Boxshall GA (2018) World of Copepods database. http://www.marinespecies.org/copepoda [accessed on 20/11/2018]

[B51] Wells JBJ (2007) An annotated checklist and keys to the species of CopepodaHarpacticoida (Crustacea). Zootaxa 1568: 1–872.

